# Newborns in crisis: An outline of neonatal ethical dilemmas in humanitarian medicine

**DOI:** 10.1111/dewb.12214

**Published:** 2018-12-26

**Authors:** Jesse Schnall, Dean Hayden, Dominic Wilkinson

**Keywords:** ethics, infant, newborn, quality of life, relief work, resource allocation

## Abstract

Newborn infants are among those most severely affected by humanitarian crises. Aid organisations increasingly recognise the necessity to provide for the medical needs of newborns, however, this may generate distinctive ethical questions for those providing humanitarian medical care. Medical ethical approaches to neonatal care familiar in other settings may not be appropriate given the diversity and volatility of humanitarian disasters, and the extreme resource limitations commonly faced by humanitarian aid missions.

In this paper, we first systematically review existing guidelines relating to the treatment and resuscitation of newborns in humanitarian crises, finding little substantive ethical guidance for those providing humanitarian healthcare. We next draw on paradigm cases and published literature to identify and describe some of the major ethical questions common to these settings. We divide these questions into quality of life considerations, allocation of limited resources, and conflicting cultural norms and values. We finally suggest some preliminary recommendations to guide ethical decision‐making around resuscitation of newborns and withdrawal of treatment in humanitarian settings.

## 1 INTRODUCTION

Humanitarian medicine presents an exceptionally challenging clinical and ethical environment for health professionals. Aid is often called for at short notice in settings of conflict, natural disaster, food insecurity, and population displacement.^1^^,^ 2 In 2016, an estimated 164.2 million people in 47 countries were in need of humanitarian assistance, with 65.6 million displaced worldwide.^3^ The ability of aid providers to meet the demands of patient care can vary dramatically in these settings. Capabilities range from gold‐standard, modern day facilities – such as those provided by United States Naval forces during the 2010 Haiti earthquake – to understaffed, basic hospitals that are rapidly inundated with patient numbers beyond their capacity.^4^^,^ 5 This burden is amplified by limited and irregular access to essential equipment supplies and resources, including operating theatres, ventilators and oxygen, medical staff, and food and running water. Missions often rely on a rotating system of short‐term volunteers, with aid providers commonly burdened by extreme working hours and insufficient sleep, and commonly enduring considerable emotional distress in light of these challenges.^6^^,^ 7

In 2014, women and children comprised over three quarters of the 84 million people in need of humanitarian assistance globally.^8^ Among these groups, neonates are disproportionately affected. Of the 15 countries with the highest neonatal mortality rates globally, 14 are characterised by chronic political instability and conflict; excluding India and China, countries experiencing such unrest account for 42% of neonatal deaths worldwide.^9^ Preterm births account for much of this burden. Reports indicate that following the Syrian Civil War, 26% of births among refugees living in Lebanon were premature, while 60% of neonatal deaths in the Zaatari refugee camp in Jordan were due to prematurity.^10^

Existing literature on ethical questions in humanitarian crises primarily discusses questions relating to the care of adults, children and pregnant women.^11^ However, neonatal care arising in these settings can result in distinctive internal and interpersonal ethical dilemmas for the humanitarian aid worker.^12^ While numerous medical guidelines exist for neonatal resuscitation, few offer specific ethical guidance on these issues in complex situations. The case examples in Box 1 illustrate some of the challenging questions faced by aid workers in humanitarian settings.^13^^,^ 14

In this paper, we first review existing guidelines relating to the treatment and resuscitation of newborns in humanitarian crises in order to determine the availability of ethical guidance for international aid workers. Second, we aim to identify and analyse some of the major ethical questions arising from the care and resuscitation of neonates during humanitarian crises that are inadequately addressed by existing guidelines, including consideration of long‐term quality of life, allocation of limited resources, and conflicting cultural norms and values between aid workers and local communities.^15^^,^ 16 ^,^ 17 There may be different ethical issues associated with different contexts (for example, acute emergency response versus ongoing medical support in settings of chronic crisis), though it is also likely that these will overlap; for the purposes of this paper, we will consider them together.^18^ In the final part of this paper, we outline some general recommendations that might provide a starting point for the development of ethical guidelines relating to the medical care of neonates in humanitarian settings.

Box 1 **Case examples of ethical questions in newborn humanitarian healthcare^19^**

1 **Asha** 20 **:**

*Two medical aid workers serving at a clinic in Haiti admit a pregnant woman, Asha, who has arrived in active labor. The fetal heart has been heard, but is very slow. The medical aid workers decide that they need to do an episiotomy to aid with delivery. The local medical doctor has supplies that will allow the medical aid workers to provide initial resuscitation of the infant. However, the clinic does not have mechanical ventilators, oxygen tanks or incubators, which will likely be needed to keep the infant alive if resuscitation is successful*.

*Given the limitations, the local doctor does not want to attempt resuscitation of the infant. She thinks that the team should prioritise the life of the mother. The medical aid workers, however, believe that they should at least attempt resuscitation and subsequently determine if the clinic can handle the infant's ongoing needs*.^21^ ***The team is unsure whether they should resuscitate a potentially compromised infant.***

**Sarah** 20:

*Sarah, a Canadian‐trained nurse deployed to provide development assistance at an urban hospital in the Caribbean, described conflicts arising in the care of premature infants:*

*“Whenever you see so many sick kids and you realize that there are some that have to be turned away, then you do say okay well we need to triage and we need to decide, you know, who we're going to treat.”*

*Premature babies were sometimes not admitted to the hospital where Sarah worked. She raised questions about the rightness of this practice, yet explained that over time she came to believe that refusing care to some was justified to ensure care for others*.^22^ ***The team must decide which premature infants should be prioritised.***

## 2 REVIEW OF LITERATURE

### 2.1 Methods

We conducted a systematic structured literature search to identify existing guidelines on the resuscitation and care of neonates in humanitarian settings.^23^ We searched databases (PubMed, Ovid MEDLINE and Google Scholar) for relevant English‐language guidelines or analyses of guidelines using a combination of relevant Medical Subject Headings (MeSH) and free text. We further searched the reference lists and citing articles of these papers for relevant articles. We also developed a list of 41 major humanitarian and medical aid agencies through this structured search.^24^ Each agency was then contacted by email with a request for relevant guidelines, while the websites and publication archives of all agencies were also searched. Guidelines were sourced and reviewed in full‐text if they related to the resuscitation and ongoing medical care of newborn infants in humanitarian settings. Material was identified that provided guidance or discussion of the specifically ethical elements of newborn care.

### 2.2 Results

Our search of published literature identified 3 articles deemed relevant to this paper as secondary references, however none included primary ethical guidelines.^25^ One of these papers, a 2010 Cochrane review, found six existing medical guidelines on perinatal and child health in crisis settings.^26^ All primary materials were sourced directly from humanitarian agencies via email or website searches. The results of agencies that replied to our email request, or had relevant publications on their websites, are available in Table 1.

**Table 1:** Existing guidelines on the resuscitation and care of neonates in humanitarian settings

Agency	Document(s)^27^	Ethical Content
UNICEF; Inter‐Agency Working Group on Reproductive Health in Crisis	Newborn Health in Humanitarian Settings (2016)^28^	No relevant ethical content
International Federation of the Red Cross (IFRC)	IFRC Maternal and Child Health Guidelines (2013)^A1^	No relevant ethical content
The UN Refugee Agency	1. Reproductive Health in Refugee Situations: An Inter‐Agency Field Manual (1999)^A2^2. Operational Guidelines on Improving Newborn Health in Refugee Situations (2013)^A3^	1. Respect for refugees’ religious and ethical values and cultural backgrounds, and provision of accessible services, privacy, confidentiality and continuity of care2. No relevant ethical content
World Health Organization (WHO)	1. WHO Manual for the Healthcare of Children in Humanitarian Emergencies (2008)^A4^2. Inter‐Agency Field Manual on Reproductive Health in Humanitarian Settings (2010)^A5^3. Essential Interventions, Commodities and Guidelines for MNCH (2011)^A6^4. Guidelines on Basic Newborn Resuscitation (2012)^A7^5. WHO Recommendations on Postnatal Care of the Newborn (2013)^A8^6. WHO Recommendations on Newborn Health (2017)^29^	1. No relevant ethical content2. Respect for communal religious and ethical values and cultural backgrounds, and universally recognized international human rights standards. Promotion of integrity, accountability and transparency in the delivery of goods and services3. No relevant ethical content4. Acknowledges the question of what constitutes ethically justified reasons to withhold resuscitation of newborns affected by high morbidity or high mortality conditions, however provides no discussion.5. No relevant ethical content6. No relevant ethical content
World Vision International	Guide to Maternal, Newborn and Child Health and Nutrition in Emergencies (2012)^A9^	Respect for human rights, humanitarian values and core operating standards, including non‐maleficence and just allocation of resources.
European Civil Protection and Humanitarian Aid Operations (ECHO)	Thematic Policy Document No.7: Health – General Guidelines (2014)^A10^	No relevant ethical content
Save the Children	Ending Newborn Deaths: Ensuring Every Baby Survives (2014)^A11^	No relevant ethical content

Few humanitarian aid agencies had developed their own medical guidelines, with some simply opting to use guidelines developed by international bodies such as the World Health Organization (WHO). Existing medical guidelines on management and resuscitation of preterm neonates were almost exclusively developed by programmes or specialised agencies of the United Nations (UN), including the WHO, the United Nations Children's Fund (UNICEF), European Civil Protection and Humanitarian Aid Operations (ECHO) and the United Nations High Commissioner for Refugees (UNHCR).

The only aid agencies with their own developed guidelines were Médecins Sans Frontières (MSF), World Vision International and Save the Children. Many of these were not specific to humanitarian settings. Ethical discussion in guidelines predominantly contained either brief references to principles such as human rights and cultural values, or did not address ethical issues, rather making blanket recommendations for resuscitation of all preterm neonates (Table 1). Of these agencies, only MSF made detailed reference to specific ethical considerations such as viability and beneficence (Table 2). However, review of the MSF guidelines indicated that they may not always be sufficiently comprehensive to guide ethical decision‐making in complex cases such as those described in Box 1. For example, while MSF guidelines emphasise adequate essential care, and consideration of the mother and child as a paired entity, more detailed guidance may be necessary on how to allocate resources between these two individuals when equipment is limited, as in the case of Asha. In the case of premature infants, MSF guidelines highlight the need for consideration of the “grey zone” of viability, medical and socio economic factors and expected long term quality of life, yet may not offer aid adequate detail on how to how to practically address the questions highlighted by Sarah's case.

**Table 2:** MSF neonatal care guidelines

Documents and Ethical Content^30^
*Essential Obstetric and Newborn Care (2015)_A12_* No relevant ethical content
***MSF Neonatal Care Policy (2016)*** 31 Neonatal care should focus on mother and child as a paired entity, and be tailored to the setting, context and level of care available, limited by medical and ethical principles. All infants determined to be viable should receive resuscitation and neonatal care, taking into consideration the pathologies of the child, the context and the medical environment. Gestational age, birth weight, clinical and neurological status, prenatal history, medical judgment and parental wishes should all be factored into decision making. Decision‐making should be based on expected long term prognosis and quality of life in the best interests of the child, taking into account mid and long term prognosis, implications for cognitive development, prevention of suffering, preservation of dignity and access to treatment. Parents should be included in the decision‐making process. Where medical interventions are destined to fail, healthcare professionals can decide to limit or stop invasive care to prevent harm. Cessation of resuscitation or limiting of treatment does not signify immediate death or abandoning of the infant.Palliation should be provided for the neonate once life saving measures are discontinued. Support to and involvement of the family is a part of this process. MSF should be attentive to quality of life, potential disabilities and short, mid and long term outcomes for neonates.
***Guidance on Limitation of Care and Palliative Care for Newborns – MSF OCG 2017*** ^**,**^ 32
Palliative care is to be provided for children with medical conditions likely to be fatal or, in cases of survival, associated with unbearable sequelae. Palliative care does not mean to stop treating or to abandon the patient, or to cause, hasten or delay death. Initiation of palliative care should be a team decision including healthcare providers and parents/legal caretakers. The choice of the family should be respected and emotional socio‐economic pressure on the family associated with their child's hospital admission should be considered. If the newborn shows severe organ dysfunction and/or severe congenital malformation, resuscitation should be ceased early. A rational treatment approach, considering limited capacities and resources in most MSF‐settings is better for the child, the parents and the clinical team. Emotional and socio‐economic pressure on the family associated with the hospital admission of their child need to be taken into account. Advanced resuscitation not to be provided for children with birthweight < 1000g or with severe congenital malformations.
**MSF International Neonatal Strategy (2017)** 33 34
Prioritisation of adequate essential care, safety, effectiveness, patient‐centeredness, beneficence and non‐maleficence Mother and baby should be considered as a paired entityM Guidance should be developed with regard to context, including religious and cultural beliefs, national legal systems, and in certain circumstances, operational constraints Care decisions should take into account expected survival without major neuro‐disability, as well as context‐specific resource limitations. Where curative care cannot be provided, emphasis should be on adequate palliative/supportive care. Interventions that are no longer beneficial, are ineffective, or will prolong the dying process without alleviating suffering should not be offered Neonatal palliative care should be provided to babies with life‐threatening conditions and uncertain prognoses. End of life (EOL) care is provided for medical conditions that are likely to be fatal, or result in severe neuro‐disability Management of preterm babies should consider the “grey zone” of viability. Decisions within this zone depend on factors such as context, resource allocation, level of care, and equipment

## 3 ETHICAL QUESTIONS

The development of specific ethical guidelines relating to newborn infants in humanitarian settings poses a serious challenge. A distinctive feature of humanitarian medical ethics is the difficulty in defining a coherent set of practices common to humanitarian medicine.^35^ Current protocols in humanitarian agencies such as MSF emphasize the lack of any single model in treating neonates, and the need to adapt guidelines on a case‐by‐case basis.^36^ This may be an advantage; even across more consistent settings (such as those relating to resuscitation of extremely premature infants in developed countries), neonatal ethical guidance has been criticized for oversimplifying complex ethical dilemmas.^37^

Others, however, argue that the absence of guidelines risks inconsistency in care, where the treatment options provided to parents can vary significantly with each treating team.^38^ An individualized approach may also create uncertainty among clinicians in navigating the complex ethical dilemmas faced during humanitarian crises.

The cases provided earlier in this paper identify some of these dilemmas. In the next section, we will outline some of the potential ethical questions, divided into three broad areas: issues relating to the patient (particularly questions over long term quality of life), issues relating to patient selection and resource allocation, and issues relating to the treating team (particularly around conflicting cultural norms and values). This is not an exhaustive list of ethical questions or dilemmas common to neonatal care in humanitarian settings. We will focus on those issues that we consider to be particularly distinctive or important for delivery of newborn humanitarian care.

### 3.1 Long‐Term Quality of Life

In the case of Asha's newborn, one potential reason to withhold resuscitation was a concern about long‐term disability if he or she survived. Considerations of long‐term wellbeing and quality of life are important to the ethics of initiating resuscitation for sick or premature newborn infants. The standard of care in developed nations is typically to withhold resuscitation only in cases where gestation, birth weight or congenital anomalies are associated with high probability of early death or unacceptably high morbidity.^39^ However, humanitarian crises commonly occur in settings where a lack of infrastructure to support children with long‐term illness or impairment may significantly alter what level of disability would be associated with mortality, constitute unacceptable morbidity or conversely, offer a reasonable quality of life.^40^

Neonates suffering from low birth weight (LBW), infection or other intrapartum events are more likely to develop long‐term adverse outcomes including cerebral palsy, cognitive problems and other neurodevelopmental disabilities.^41^ While early interventions such as drying, immediate stimulation, thermal care and Bag‐Mask Ventilation (BMV) may reduce the likelihood, severity and cost of future disability for at‐risk neonates, they may also prolong the lives and associated costs of severely disabled infants who would otherwise not have survived. A Burundi‐based study of LBW infants found that 27% had one or more impairments on 2‐year follow up from birth.^42^

In low resource settings where support options are lacking, the consequences of such conditions are likely to be more severe not only for the infant, but also their family.^43^ In many Low and Middle‐Income Countries (LMICs), healthcare costs are primarily paid for out of pocket.^44^ In severe cases, financial debt resultant from neonatal care can push families into acute nutritional crises and contribute to the death of other family members.^45^ International aid workers from developed nations often cite economic costs upon society and lifelong stress imposed on the family as reasons not to resuscitate infants at high risk of disability.^46^^,^ 47 The same Burundi‐based study of LBW infants found that 7% of children were reported to be an additional burden to the family as a result of their impairments.^48^

Humanitarian crises involving physical dislocation and migration present a serious obstacle to necessary follow‐up and monitoring of unwell infants. Even in the absence of such destabilisation, humanitarian aid missions may only be stationed in disaster regions for short, fixed periods.^49^ Aid workers may therefore be unable to provide a sufficiently comprehensive process of care to ensure acceptable quality of life.

Impairments may also be a source of considerable social stigma in developing regions. Neurocognitive disabilities may lead to the removal of affected children from their schooling, while families in some settings have been shown to react poorly when faced with a handicapped or impaired baby.^50^^,^ 51 Stigma and familial rejection carry significant potential to detrimentally impact the neonate's quality of life, and the extent of discrimination against individuals with impairments can vary significantly depending on cultural or religious norms.

Attention to the interests and wellbeing of Asha's infant (Box 1) might suggest that resuscitation should be provided. However, even if treatment would be in the interests of the infant, there is a further ethical question about the impact of treatment on others.

### 3.2 Resource Allocation

Resource scarcity is a significant concern for providers of humanitarian aid.^52^ Missions are frequently faced with situations in which available resources are insufficient to sustain life, or inadequate to meet patient needs.^53^ As exemplified by the case of Sarah (Box 1), the choices presented by a lack of essential resources create a recurring ethical struggle for aid providers, who must wrestle with distributive justice challenges in their capacity to provide care.^54^

There are different levels of resource allocation in humanitarian missions. For example intervention choices between the needs of distinct populations (macro), different programmatic needs within a population (meso), and varying individual patient needs (micro).^55^ Questions relating to newborn infants, are most likely to arise at the meso or micro‐levels of humanitarian resource allocation (although these will be affected by decisions at the macro‐level).

In the past, newborn infants have sometimes been regarded as a low priority for medical care in humanitarian missions.^56^ Resuscitation of newborns, particularly preterm infants and those with birth complications, can be heavily demanding on both the number of staff and the time required for treatment. Providing resuscitation may impede care for other patients by reducing availability of the treating team. Irregular deliveries of medicines and limited access to equipment force treating teams to conserve resources in consideration of future patient need. Successfully resuscitated neonates are likely to be highly vulnerable and require more extensive care. They are also completely financially dependent on families and aid missions for the funding of any treatment.

One important consideration for humanitarian missions is the need to achieve the greatest health benefit for those in need of their support. Agencies such as MSF emphasise the provision of essential care as a means to saving the most lives.^57^ One way of maximizing benefit would be to compare different health interventions and to selectively provide those that are most cost‐effective.^58^ However, it is challenging to assess the relative impact of providing neonatal care. The feasibility of collecting neonatal health data in volatile, varied humanitarian disasters is questionable, with many current methods fraught with errors and limitations.^59^ The Burundi study of LBW infants found that over one quarter were lost to follow up, due to reasons including death, familial migration, and estrangement from community figures.^60^

Studies from Mexico and Zambia have highlighted the cost‐effectiveness of neonatal intensive care and neonatal resuscitation training respectively in LMIC settings.^61^^,^ 62 Extant data also suggests that including neonatal interventions in home‐based care packages in LMIC settings would increase overall cost‐effectiveness of care.^63^ However, existing data sets are frequently built upon uncertain assumptions and extrapolations which, even if accurate, might not be true of humanitarian crises.^64^^,^ 64

Prioritisation of children or young adults over preterm infants may lead to justice concerns. The guiding rationale of clinical triage often reflects egalitarian principles, rather than strictly utilitarian ones.^65^ Alternatively, selective provision of treatment to newborns may conflict with the values of communities. Care of the mother, or of other non‐neonate patients, may be more desirable for local communities, depending on the value they place on saving newborns.^66^ (That might be one explanation for the desire to focus medical attention on Asha rather than her infant (Box 1)). One important consideration for aid agencies will be how or whether to reflect local cultural norms in their delivery of healthcare.

### 3.3 Competing Cultural Norms and Values

International aid workers may face myriad cultural differences and challenges during humanitarian crises that are of distinctive importance in the context of neonatal care.^67^ These may include conflicting religious and traditional beliefs regarding the moral status of the neonate; divergent perceptions of the roles of parents and treating teams with regard to medical decision making; and lower levels of health literacy than is common in Western nations. The ability to understand, communicate and navigate these differences presents a significant challenge to aid workers, who may be required to act in opposition to their personal views and training in order to meet the expectations of local communities and parents.

The LMICs which often play host to humanitarian disasters are likely to exhibit a range of attitudes toward neonates, particularly those who are preterm or disabled. Reflections from Cameroon promote the unconditional acceptance and moral worthiness of the neonate, irrespective of disability or gender.^68^ Conversely, perspectives from countries such as India indicate that neonates may be viewed as mere additions to the family structure, rather than individual persons with distinct moral and legal rights.^69^ Many communities worldwide do not consider a birth as ‘complete’ until the infant has survived the dangerous initial neonatal period. It is only after this period that the infants are named and recognized as distinct individuals.^70^ Religious beliefs may also be of greater significance than is common in many secular democracies in the developed world, often playing a significant role during decisions regarding neonatal care and the withholding of treatment and resuscitation.^71^^,^ 72

Attitudes of parents and physicians in LMICs may be far more heavily influenced by potential disability, as well as the gender of the neonate. A survey of Mongolian healthcare providers found that fewer than half felt that newborns with birth‐defects would be accepted as normal in society.^73^ Attitudes among physicians in India reflect a similar degree of discrimination towards disabled infants, with an observed preference to only discharge healthy babies.^74^ Disabled children commonly experience significant individual and social neglect in Indian society, while female babies may also be stigmatised.^75^ Families are often less willing to pay for intensive care and medicines for female infants, or to attend follow‐up consultations.^76^

Cultural differences may also influence how ethically fraught decisions are approached. In Western societies, it is common for parents to make decisions alongside the treating staff, with an overwhelming majority of health‐care providers believing parents should have the final say in their infant's care.^77^ Such collaborative approaches may not be the norm, or may not be as practicable, in humanitarian crises. Providers in some settings may be required to include elder family members in treatment decisions.^78^ Aid workers may also be required to navigate exclusionary gender roles when communicating with parents.^79^

In other arenas, doctors may be expected to adopt complete responsibility for decision‐making. Such approaches are common in some countries, in which it is typically considered unfair to impose responsibility on the parents of the child.^80^^,^ 81 Parents who are poorly educated or from low socioeconomic backgrounds may be unable to understand medical terms or comprehend the seriousness of potential disability for the neonate, impairing their potential for involvement in the decision‐making process.^82^

## 4 DEVELOPING ETHICAL GUIDANCE RELATING TO NEWBORN HUMANITARIAN CARE

The distinctive ethical question of when to resuscitate neonates is complicated by the volatility, resource limitations and cultural, geographical and situational diversity of humanitarian aid settings. Development of detailed ethical guidelines and policies specific to neonatal care is necessary for the provision of considerate, consistent and effective care.^83^

Our review of existing literature identified little current guidance relating to neonatal ethical dilemmas in humanitarian crises. In the previous section, we aimed to identify and outline the distinctive ethical questions in this field. It is not possible in the space of this short paper to provide definitive answers to these challenging questions. Nevertheless, some preliminary conclusions or suggestions may be worth highlighting.

### 4.1 Resuscitation and Stabilization

While resource allocation in humanitarian crises is ethically fraught and challenging, prioritization of simple, cost‐effective interventions for the care of newborns should be uncontroversial. Within hospitals, delayed cord clamping for 60 seconds or more may be of distinct value for infants with respiratory difficulties by providing placental transfusion.^84^ Use of room air as a default for neonatal resuscitation is perhaps uniquely cost‐effective in being technically easier and cheaper (than resuscitation in oxygen) and likely resulting in improved outcomes.^85^^,^ 86 ^,^ 87

Where hospital admission or intensive care are limited by resource concerns, aid workers such as Sarah should be encouraged to either provide, or educate mothers and care‐givers on early interventions such as drying, immediate stimulation, airway clearing where indicated, thermal care, suction and Bag‐Mask Ventilation (BMV). These interventions can reduce a large proportion of perinatal deaths with little cost.^87^^,^ 88 Additional simple measures such as breastfeeding support, infection prevention and management, and kangaroo mother care may be valuable and should be promoted even if there is little capacity to provide more intense medical treatment for preterm infants.^89^ A lack of the highest level care should not be equated with no care at all.

Questions of which preterm infants to resuscitate (highlighted by Sarah's case) are sometimes answered using gestational age (GA) based guidelines. In developed nations, prior to 22 weeks, resuscitation is almost never recommended, while at 25 weeks, it is usually mandated.^90^ The period in between has been termed the ‘grey zone,’ during which the choice between resuscitation and palliation is guided by parental wishes.^91^

A similar grey zone might be considered for humanitarian missions, with upper and lower bounds based on locally relevant data on the outcome for preterm infants with available technology. However, there are likely to be some differences, and challenges.

First, in humanitarian crises, GA‐based guidelines may not be readily applicable or appropriate. In LMICs, gestational ages are unlikely to be known with certainty because of a lack of early obstetric ultrasound and uncertain menstrual dates.^92^ Birth weight might be used instead, however, aid workers may not have any locally relevant outcome data necessary to develop guidelines. Infants who require invasive respiratory support are not likely to be treatable, even at more viable GAs or birthweight.

Second, the grey zone may be shifted upward (i.e. to a higher gestational age/weight) in humanitarian crises due to resource limitations. For preterm infants who might elsewhere be expected to have a good outcome, lack of resources may require treating teams to not resuscitate on grounds of distributive justice.

Third, the grey zone may be widened due to a need to give greater weight to the wishes of parents. In non‐humanitarian settings, treating teams may act against the wishes of parents through legal channels. A survey of South African neonatal intensive care units showed that 93% of physicians would resuscitate moderately premature infants (28‐29 weeks GA) despite parental refusal.^93^ However, legal recourse is unlikely to exist, or be practical, in humanitarian environments. Resuscitation against the wishes of the parents will likely cause significant distress and mistrust. Families may simply abandon, or be unable to care for the infant, due to financial limitations or cultural beliefs.^94^ Aid missions are poorly positioned to engage in care or adoption of abandoned infants, and may lack the time and resources to liaise with potentially inundated local adoption services.

### 4.2 Withdrawal of Treatment and Palliative Care

In circumstances where the outcome of resuscitation is uncertain, as in the case of Asha's infant, aid workers might consider commencing resuscitation (assuming availability of necessary resources), with subsequent monitoring and assessment. Following resuscitation, review and assessment of the neonate's survival prospects and likely quality of life should guide treating teams on whether to continue active treatment or to shift to palliative care. Decisions should be guided by concern for the infant's best interests, with attention to both quality and quantity of life, and availability of resources.^95^ MSF guidelines on the scope of neonatal care stress consideration of the likelihood of ongoing neurodisability in the event of successful treatment, emphasizing the requirement to do no harm.^96^ Intensive care may therefore not be a viable option for infants requiring complex or long‐term treatment for which existing resources are insufficient.

Treatment decisions should also be guided by the UN Convention on the Rights of the Child, with particular concern paid to articles 23 and 27 (Article 23 recognises the right of a mentally or physically disabled child to enjoy a full and decent life with access to extensive care and education subject to available resources. Article 27 recognises the right of every child to a standard of living adequate for their full development and the responsibilities of the parent (or others) to provide satisfactory living conditions for the child, within their means and with any necessary assistance from the state).^97^ If the potential for long‐term disability remains high following initial resuscitation, and parents, local communities and governments are unable to guarantee the ongoing care necessary for the development and wellbeing of the neonate, withdrawal of treatment and initiation of palliative care may be indicated. Health care workers should advocate for the interests of the child, however, may have limited options to provide treatment against the wishes of parents.

In the case of Asha's infant, the treating team must consider the likelihood of survival with a reasonable quality of life in that setting, and the availability of treatment for the infant. Challenges include uncertainties in the infant's outcome with treatment, the level of treatment the infant would require after initial resuscitation and the capability of Asha's family to provide ongoing care in the event of long‐term disability resulting from complications. Given those uncertainties, it may be warranted to provide resuscitation in the first instance, with a plan to withdraw treatment and palliate if the infant were to require ongoing respiratory support. If treatment of the infant would compromise the treatment of their mother, the latter should be prioritised.

### 4.3 Navigating Cultural Norms and Values

Cultural and religious differences, historical conflict and mistrust, greater patient vulnerability and linguistic barriers must be navigated with care in the humanitarian environment. Given the variation in values not only between countries but also individual communities, aid agencies should train and educate international workers in the local beliefs and customs of their setting of work on a case‐by‐case basis to ensure adequate sensitivity and understanding of the unique norms and values in that setting. This does not imply cultural relativism in the provision of medical care. Rather, it is a need to reflect the contextual nature of ethical decision‐making. Local belief systems greatly influence approaches to life and death, and must be a part of conversations with families.^98^ Initial and ongoing consultation with communal and spiritual leaders may be indicated, particularly when agencies are uncertain of local beliefs and customs.

Aid providers must be flexible with regard to the decision‐making process, and should also be encouraged to seek out and consider the wishes of extended family and community leaders where appropriate when weighing treatment options. Collaboration with local translators, medical staff and educated community members may be necessary to establish productive relationships with parents and the community, and aid communication of complex medical information to ensure maximum inclusion of parents in treatment decisions. However, such flexibility also has its limits. Rarely, it may be necessary for aid workers to oppose parental wishes or locally held beliefs in order to safeguard the best interests of the infant, or enable provision of care to other infants. Clear communication of the reasons for this opposition will be important in such circumstances.

## 5 CONCLUSION

Humanitarian medical care is logistically, technically and ethically complex. Extension of humanitarian medicine to include newborn infants raises additional challenges for those providing and organizing humanitarian medical care. We have reviewed existing guidelines that inform medical care in such settings, described some of the ethical challenges, and suggested key ethical principles that can be applied to neonatal care decisions in these settings. Prioritisation of simple, cost‐effective interventions for the management of the neonate should be encouraged. Widening and upward shifting of the grey zone of neonatal resuscitation may be necessary given likely uncertainties over gestational ages and birth weights, and limitations in resources and local legal protocols. Decisions over whether to continue treatment or initiate palliative care should be guided by the infant's best interests, with particular focus on likely quality and quantity of life in the local setting, as well as resource availability. Familial and communal wishes and customs may also take on greater weight in humanitarian settings, and must be navigated with care. While these principles provide a starting point for the aid worker, further empirical and analytical research will be vital to help offer guidance to those providing neonatal medical care in the setting of humanitarian aid.

## FUNDING

Dominic Wilkinson was supported for this work by a grant from the Wellcome Trust WT106587/Z/14/Z.

## CONFLICT OF INTEREST STATEMENT

The authors declare they have no conflict of interest interests.

## Notes

1 Toole M, Waldman R. The public health aspects of complex emergencies and refugee situations. *Annu Rev Public Health*. 1997;18(1):283‐312.

2 Lam JO, Amsalu R, Kerber K, et al. Neonatal survival interventions in humanitarian emergencies: A survey of current practices and programs. *Confl Health*. 2012;6(1):2.

3 Lattimer C, Swithern S. Gobal humanitarian assistance report 2017. United Kingdom: Development Initiatives Ltd.; c2017 [cited 2017 Oct 27]. Available from: http://devinit.org/wp-content/uploads/2017/06/GHA-Report-2017-Full-report.pdf.

4 Walk RM, Donahue TF, Sharpe RP, Safford SD. Three phases of disaster relief in Haiti—pediatric surgical care on board the United States naval ship comfort. *J Pediatr Surg*. 2011;46(10):1978‐1984.

5 Mulvey JM, Awan SU, Qadri AA, Maqsood MA. Profile of injuries arising from the 2005 Kashmir earthquake: The first 72h. *Injury*. 2008;39(5):554‐560.

6 Ibid

7 Burnweit C, Stylianos S. Disaster response in a pediatric field hospital: Lessons learned in Haiti. *J Pediatr Surg*. 2011;46(6):1131‐1139.

8 Zeid S, Gilmore K, Khosla R, et al. Women's, children's, and adolescents’ health in humanitarian and other crises. *Br Med J*. 2015;351.

9 Wise PH, Darmstadt GL. Confronting stillbirths and newborn deaths in areas of conflict and political instability: A neglected global imperative. *Paediatr Int Child Health*. 2015;35(3):220‐226.

10 DeJong J, Ghattas H, Bashour H, et al. Reproductive, maternal, neonatal and child health in conflict: A case study on Syria using countdown indicators. *BMJ Glob Health*. 2017;2(3).

11 Schwartz L, Sinding C, Hunt M, et al. Ethics in humanitarian aid work: Learning from the narratives of humanitarian health workers. *AJOB Prim Res*. 2010;1(3):45‐54.

12 Lattimer C, Swithern S, op. cit. note 3.

13 Schwartz L, Sinding C, Hunt M, et al., op. cit. note 11.

14 Wall AE. *Ethics for international medicine: A practical guide for aid workers in developing countries*. Hanover: Dartmouth College Press; 2012. p.58.

15 Iserson KV, Biros MH, James Holliman C. Challenges in international medicine: Ethical dilemmas, unanticipated consequences, and accepting limitations. *Acad Emerg Med*. 2012;19(6):683‐692.

16 Medecins Sans Frontieres International Pediatrics Working Group. MSF international neonatal strategy. Geneva: MSF; c2017 [cited 2017 Dec 10].

17 Miljeteig I, Sayeed SA, Jesani A, Johansson KA, Norheim OF. Impact of ethics and economics on end‐of‐life decisions in an Indian neonatal unit. *Pediatrics*. 2009;124(2):e322.

18 Calain P. In search of the ‘new informal legitimacy’ of Médecins Sans Frontières. *Public Health Ethics*. 2012;5(1):56‐66. 

19 Adapted from original sources.

20 Pseudonym

21 Wall AE, op. cit. note 14.

22 Schwartz L, Sinding C, Hunt M, et al., op. cit. note 11.

23 A full description of the search strategy is available in Appendix 1

24 A full list of the agencies contacted is available in Appendix 2.

25 A full description of the search results is available in Appendix 1

26 Turner TJ, Barnes H, Reid J, Garrubba M. Evidence for perinatal and child health care guidelines in crisis settings: Can Cochrane help? *BMC Public Health*. 2010;10:170.

27 For references A1‐A11, see appendix 3.

28 United Nations Children’s Fund. Newborn health in humanitarian settings: Field guide (interim version). New York: UNICEF; c2016 [cited 2017 Nov 8]. Available from: http://www.unicefinemergencies.com/downloads/eresource/docs/Health/NewBornHealthBook-ProductionV17-Web.pdf.

29 World Health Organization. WHO recommendations on newborn health. Geneva: WHO; c2017 [cited 2017 Nov 11]. Available from: http://apps.who.int/iris/bitstream/handle/10665/259269/WHO-MCA-17.07-eng.pdf?sequence=1.

30 For references A12‐A13, see appendix 3.

31 Provided by personal correspondence from Philippe Calain, MSF.

32 Medecins Sans Frontieres.

33 Provided by personal correspondence from Philippe Calain, MSF.

34 Medecins Sans Frontieres International Pediatrics Working Group, op. cit. note 16.

35 Calain P, op. cit. note 18

36 Medecins Sans Frontieres, op. cit. note 32.

37 Janvier A, Leblanc I, Barrington KJ. The best‐interest standard is not applied for neonatal resuscitation decisions. *Pediatrics*. 2008;121(5):963‐969.

38 Wilkinson D, Savulescu J. Disability, discrimination and death: Is it justified to ration life saving treatment for disabled newborn infants? *Monash Bioeth Rev*. 2014;32(1‐2):43‐62.

39 Chadha IA. Neonatal resuscitation: Current issues. *Indian J Anaesth*. 2010;54(5):428‐438.

40 Miljeteig I, Sayeed SA, Jesani A, Johansson KA, Norheim OF, op. cit. note 17.

41 Blencowe H, Vos T, Lee ACC, et al. Estimates of neonatal morbidities and disabilities at regional and global levels for 2010: Introduction, methods overview, and relevant findings from the global burden of disease study. *Pediatr Res*. 2013;74(Suppl 1):4‐16.

42 van den Boogaard W, Zuniga I, Manzi M, et al. How do low‐birthweight neonates fare 2 years after discharge from a low‐technology neonatal care unit in a rural district hospital in Burundi? *Trop Med Int Health*. 2017;22(4):423‐430.

43 Ibid

44 Xu K, Evans DB, Carrin G, et al. Protecting households from catastrophic health spending. *Health Aff (Millwood)*. 2007;26(4):972‐983.

45 Miljeteig I, Johansson KA, Sayeed SA, Norheim OF. End‐of‐life decisions as bedside rationing. An ethical analysis of life support restrictions in an Indian neonatal unit. *J Med Ethics*. 2010;36(8):473.

46 Eden LM, Callister LC. Parent involvement in end‐of‐life care and decision making in the newborn intensive care unit: An integrative review. J Perinat Educ. 2010;19(1):29‐39.

47 Miljeteig I, Sayeed SA, Jesani A, Johansson KA, Norheim OF, op. cit. note 17.

48 van den Boogaard W, Zuniga I, Manzi M, et al., op. cit. note 42.

49 Burnweit C, Stylianos S, op. cit. note 7.

50 Miljeteig I, Sayeed SA, Jesani A, Johansson KA, Norheim OF, op. cit. note 17.

51 World Health Organization. Neurological disorders: Public health challenges. Geneva: WHO; c2006 [cited 2017 Oct 22]. Available from: http://www.who.int/mental_health/publications/neurological_disorders_ph_challenges/en/.

52 Medecins Sans Frontieres International Pediatrics Working Group, op. cit. note 16.

53 Schwartz L, Sinding C, Hunt M, et al. op. cit. note 11.

54 Ibid

55 Ford N, Zachariah R, Mills E, Upshur R. Defining the limits of emergency humanitarian action: Where, and how, to draw the line? *Public Health Ethics*. 2010;3(1):68‐71.

56 Personal correspondence, Philippe Calain.

57 Medecins Sans Frontieres International Pediatrics Working Group, op. cit. note 16.

58 Formal cost‐effectiveness analysis has not traditionally been a major consideration for humanitarian organisations like MSF. Personal correspondence, Philippe Calain.

59 Turner EL, Nielsen KR, Jamal SM, von Saint André‐von Arnim A, Musa NL. A review of pediatric critical care in resource‐limited settings: A look at past, present, and future directions. *Front Pediatr*. 2016;4:5.

60 van den Boogaard W, Zuniga I, Manzi M, et al., op. cit. note 42.

61 Profit J, Lee D, Zupancic JA, et al. Clinical benefits, costs, and cost‐effectiveness of neonatal intensive care in Mexico. *PLoS Med*. 2010;7(12):e1000379.

62 Sabin LL, Knapp AB, MacLeod WB, et al. Costs and cost‐effectiveness of training traditional birth attendants to reduce neonatal mortality in the Lufwanyama neonatal survival study (lunesp). *PLoS ONE*. 2012;7(4):e35560.

63 LeFevre AE, Shillcutt SD, Waters HR, et al. Economic evaluation of neonatal care packages in a cluster‐randomized controlled trial in Sylhet, Bangladesh. *Bull World Health Organ*. 2013;91:736‐745.

64 Woods B, Revill P, Sculpher M, Claxton K. Country‐level cost‐effectiveness thresholds: Initial estimates and the need for further research. *Value Health*. 2016;19(8):929‐935.

65 Medecins Sans Frontieres International Pediatrics Working Group, op. cit. note 16.

66 Hayden D, Wilkinson D. Asymmetrical reasons, newborn infants, and resource allocation. *Am J Bioeth*. 2017;17(8):13‐15.

67 Iserson KV, Biros MH, James Holliman C., op. cit. note 15.

68 Tangwa GB. The traditional African perception of a person: Some implications for bioethics. *Hastings Cent Rep*. 2000;30(5):39‐43.

69 Miljeteig I, Johansson KA, Sayeed SA, Norheim OF, op. cit. note 45.

70 World Health Organization. Neonatal and perinatal mortality: Country, regional and global estimates. Geneva: WHO; c2006 [cited 2017 Nov 10]. Available from: http://apps.who.int/iris/bitstream/10665/43444/1/9241563206_eng.pdf.

71 McAdams RM, Erdenebileg A, Batra M, Gerelmaa Z. Attitudes of healthcare providers towards non‐initiation and withdrawal of neonatal resuscitation for preterm infants in Mongolia. *J Health Popul Nutr*. 2012;30(3):346‐352.

72 Miljeteig I, Norheim OF. My job is to keep him alive, but what about his brother and sister? How Indian doctors experience ethical dilemmas in neonatal medicine. *Dev World Bioeth*. 2006;6(1):23‐32.

73 McAdams RM, Erdenebileg A, Batra M, Gerelmaa Z, op. cit. note 72.

74 Miljeteig I, Sayeed SA, Jesani A, Johansson KA, Norheim OF, op. cit. note 17.

75 Miljeteig I, Johansson KA, Sayeed SA, Norheim OF, op. cit. note 45.

76 Eden LM, Callister LC, op. cit. note 46.

77 Miljeteig I, Sayeed SA, Jesani A, Johansson KA, Norheim OF, op. cit. note 17.

78 Dumbaugh M, Tawiah‐Agyemang C, Manu A, et al. Perceptions of, attitudes towards and barriers to male involvement in newborn care in rural Ghana, West Africa: A qualitative analysis. *BMC Pregnancy and Childbirth*. 2014;14(1):269.

79 da Costa DE, Ghazal H, Al Khusaiby S. Do not resuscitate orders and ethical decisions in a neonatal intensive care unit in a Muslim community. *Arch Dis Child Fetal Neonatal Ed*. 2002;86(2):F115.

80 Miljeteig I, Norheim OF, op. cit. note 73.

81 Ibid

82 Ersdal HL, Singhal N. Resuscitation in resource‐limited settings. *Semin Fetal Neonatal Med*. 2013;18(6):373‐378.

83 Ibid

84 Saugstad OD, Ramji S, Vento M. Resuscitation of depressed newborn infants with ambient air or pure oxygen: A meta‐analysis. *Neonatology*. 2005;87(1):27‐34.

85 Tan A, Schulze AA, O'Donnell CPF, Davis PG. Air versus oxygen for resuscitation of infants at birth. *Cochrane Database Syst Rev*. 2005(2).

86 Davis PG, Tan A, O'Donnell CPF, Schulze A. Resuscitation of newborn infants with 100% oxygen or air: A systematic review and meta‐analysis. *Lancet*. 2004;364(9442):1329‐1333.

87 Ersdal HL, Singhal N, op. cit. note 84.

88 World Health Organization, op. cit. note 90.

89 United Nations Children’s Fund, op. cit. note 28.

90 Guillén Ú, Weiss EM, Munson D, et al. Guidelines for the management of extremely premature deliveries: A systematic review. *Pediatrics*. 2015;136(2):343.

91 Wilkinson D. The grey zone in neonatal treatment decisions. In *When doctors and parents disagree: Ethics, paediatrics and the zone of parental discretion*. Ed. McDougall R, Delany C, Gillam L. Annandale, NSW: The Federation Press; 2016.

92 Lee ACC, Katz J, Blencowe H, et al. National and regional estimates of term and preterm babies born small for gestational age in 138 low‐income and middle‐income countries in 2010. *Lancet Global Health*. 2013;1(1):e26‐e36.

93 Partridge JC RT, Ballot DE, Martinez AM, Cory BJ, Davies VA. Intensive care for very low birthweight infants in South Africa: A survey of physician attitudes, parent counseling and resuscitation practices. *J Trop Pediatr*. 2005;51(1):11‐16.

94 Turner EL, Nielsen KR, Jamal SM, von Saint André‐von Arnim A, Musa NL, op. cit. note 59.

95 Larcher V. Ethical considerations in neonatal end‐of‐life care. *Semin Fetal Neonatal Med*. 2013;18(2):105‐110.

96 Medecins Sans Frontieres International Pediatrics Working Group, op. cit. note 16.

97 United Nations High Commissioner for Refugees. Convention on the rights of the child. New York: UNHCR; c1989 [cited 2018 Jun 20]. Available from: http://www.unhcr.org/en-au/protection/children/50f941fe9/united-nations-convention-rights-child-crc.html.

98 Turner EL, Nielsen KR, Jamal SM, von Saint André‐von Arnim A, Musa NL, op. cit. note 59.

