# Supplementary material for: Newborns in crisis: An outline of neonatal ethical dilemmas in humanitarian medicine
Source: Dev World Bioeth. 2018 Dec 26;19(4):196–205. doi: 10.1111/dewb.12214 (PMC6916396; doi:10.1111/dewb.12214)
Supplement: Supplementary file 1 [file DEWB-19-196-s001.docx]

Appendix 1 – Search Strategy and Terms

We searched databases (PubMed, Ovid MEDLINE and Google Scholar) using a combination of the following subject headings (Medical Subject Headings) and free text:

(preterm infants; OR preterm birth; OR low gestational age; OR extremely low gestational age; OR extremely low birth weight; OR limit of viability; OR neonate; OR infant; OR newborn) AND (humanitarian; OR humanitarian aid; OR humanitarian disaster; OR humanitarian relief; OR relief agencies; OR humanitarian settings) AND (practice guidelines; OR clinical practice guidelines).

No language restrictions were applied.

We identified 2 results on PubMed and 8 results on Ovid MEDLINE; of these 10 articles, 3 were duplicates, leaving 7 unique papers. Of the 7 distinct articles, 3 were deemed relevant to this paper as secondary references, however none included primary technical or ethical guidelines.

We also searched the reference lists and citing articles of these papers, none of which yielded any primary materials. Further searches on Google scholar yielded relevant results in the literature, but no primary materials.
