# Supplementary material for: Newborns in crisis: An outline of neonatal ethical dilemmas in humanitarian medicine
Source: Dev World Bioeth. 2018 Dec 26;19(4):196–205. doi: 10.1111/dewb.12214 (PMC6916396; doi:10.1111/dewb.12214)
Supplement: Supplementary file 2 [file DEWB-19-196-s002.docx]

Appendix 2 – List of Humanitarian and International Organisations Contacted

Action Against Hunger

African Medical and Research Foundation (AMREF)[1]

American Refugee Committee/ARC International

AmeriCares

Association of Medical Doctors of Asia

CARE

Caritas

Center for Health Policy and Innovation

Centre for Operations Research and Training

CHS Alliance

Cooperativa MLO

Cooperazione Internazionale (COOPI)

DARA (international organization)

Doctors of the World

Doctors Without Borders

ECHO (European Commission)

Inter Agency Working Group on Reproductive Health Crisis

International Committee of the Red Cross

International Federation of the Red Cross

International Medical Corps

International Rescue Committee

Islamic Relief

IsraAid

Jugend Eine Welt

Mae Tao Clinic

Malteser International

Medair

Médecins du Monde

Mercy Corps

Nas Foundation

Oxfam

Plan International

Samaritan's Purse (world Medical Mission)

Save the Children

The UN Refugee Agency (UNHCR)

The United Nations Children’s Fund (UNICEF)

United Nations Population Fund (UNFPA)

White Ribbon Alliance for Safe Motherhood

World Health Organization (WHO)

World Concern

World Vision International
