# Supplementary material for: Newborns in crisis: An outline of neonatal ethical dilemmas in humanitarian medicine
Source: Dev World Bioeth. 2018 Dec 26;19(4):196–205. doi: 10.1111/dewb.12214 (PMC6916396; doi:10.1111/dewb.12214)
Supplement: Supplementary file 3 [file DEWB-19-196-s003.docx]

Appendix 3 – Table 1 & 2 Citations

A1. International Federation of the Red Cross. Maternal, newborn and child health framework. Geneva: IFRC; c2013 [cited 2017 Oct 15]. Available from: [http://www.ifrc.org/PageFiles/93927/1232600-MNCH Framework report_LR (2).pdf](http://www.ifrc.org/PageFiles/93927/1232600-MNCH%20Framework%20report_LR%20(2).pdf).

A2. United Nations High Commissioner for Refugees. Reproductive health in refugee sitautions. An inter-agency field manual. New York: UNHCR; c1999 [cited 2017 Oct 19]. Available from: <http://www.refworld.org/docid/403b6ceb4.html>

A3. United Nations High Commissioner for Refugees. Operational guidelines on improving newborn health in refugee operations. New York: UNHCR; c2013 [cited 2017 Oct 19]. Available from: <http://www.unhcr.org/en-au/protection/health/54bd0dc49/operational-guidelines-improving-newborn-health-refugee-operations.html>.

A4. World Health Organization. Manual for the health care of children in humanitarian emergencies. Geneva: WHO; c2008 [cited 2017 Oct 19]. Available from: <http://apps.who.int/iris/bitstream/10665/43926/1/9789241596879_eng.pdf>.

A5. World Health Organization. Inter-agency field manual on reproductive health in humanitarian settings. Geneva: WHO; c2010 [cited 2017 Oct 19]. Available from: <http://www.who.int/reproductivehealth/publications/emergencies/field_manual/en/>.

A6. World Health Organization. Essential interventions, commodities and guidelines for reproductive, maternal, newborn and child health. Geneva: WHO; c2011 [cited 2017 Oct 18]. Available from: <http://www.who.int/entity/pmnch/topics/part_publications/essential_interventions_18_01_2012.pdf?ua=1>.

A7. World Health Organization. Guidelines on basic newborn resuscitation. Geneva: WHO; c2012 [cited 2017 Oct 20]. Available from: <http://www.who.int/maternal_child_adolescent/documents/basic_newborn_resuscitation/en/>.

A8. World Health Organization. Who recommendations on postnatal care of the mother and newborn. Geneva: WHO; c2013 [cited 2017 Oct 18]. Available from: <http://www.who.int/maternal_child_adolescent/documents/postnatal-care-recommendations/en/>.

A9. World Vision International. Guide to maternal, newborn and child health and nutrition in emergencies. California: World Vision International; c2012 [cited 2017 Oct 19]. Available from: [https://www.wvi.org/sites/default/files/MNCH in Emergencies Guide FINAL_2.pdf](https://www.wvi.org/sites/default/files/MNCH%20in%20Emergencies%20Guide%20FINAL_2.pdf).

A10. European Civil Protection and Humanitarian Aid Operations. Thematic policy document no.7: Health – general guidelines. Brussels: ECHO; c2014 [cited 2014 Oct 20]. Available from: <http://ec.europa.eu/echo/files/policies/sectoral/health_thematic_policy_document_en.pdf>.

A11. Save The Children. Ending newborn deaths: Ensuring every baby survives. London: Save The Children; c2014 [cited 2017 Oct 20]. Available from: <https://www.savethechildren.net/sites/default/files/libraries/ENDING-NEWBORN-DEATHS.pdf>.

A12. Medecins Sans Frontieres. Essential obstetric and newborn care Paris: MSF; c2015 [cited 2017 Oct 21]. Available from: <http://refbooks.msf.org/msf_docs/en/obstetrics/obstetrics_en.pdf>.

A13. Medecins Sans Frontieres International Pediatrics Working Group. Msf international neonatal strategy. Geneva: MSF; c2017 [cited 2017 Dec 10].
